# Assessment and practical science: identifying generalizable characteristics of written assessments that reward and incentivise effective practices in practical science lessons

**DOI:** 10.1080/09500693.2023.2253366

**Published:** 2023-11-19

**Authors:** Alistair M. Moore, Peter Fairhurst, Judith M. Bennett, Christine Harrison, Catarina F. Correia, Jessie Durk

**Affiliations:** a Department of Education, University of York, UK; b School of Education, Communication & Society, King’s College London, UK

**Keywords:** Assessment, practical work, washback

## Abstract

High-stakes assessments prominently influence what is done in secondary school science lessons (‘washback’ effects). It is therefore important that assessments of knowledge and understanding gained from practical work are constructed to reward and incentivise effective practices in practical work. To do that, they must differentiate between pupils who have experienced practical work in different ways. This empirical, mixed-methods study identifies generalizable characteristics of written assessments that differentially reward pupils who experienced practical activities through hands-on work, teacher demonstration, video demonstration, or reading about the activity. Conclusions are drawn from 1486 post-intervention tests completed by pupils aged 14–15 in England, from lesson observations and teacher interviews. This study also identifies pedagogical practices that were more noticeable in practical work that was most rewarded by the written assessments: the work was teacher-guided; and pupils were encouraged to be active participants. Existing literature describes negative washback effects of high-stakes, written assessments that limit the use and effectiveness of practical work as a pedagogical tool. We describe ways in which written assessments could be constructed to better reward effective practices in practical work (practices that better support learning), with the intention of having positive washback effects on pedagogy by better incentivising these practices.

## Introduction

### Purposes of practical work

The school science curricula of various countries require pupils to engage in activities that combine the manipulation of real objects, materials and apparatus with various amounts of observation, measurement, experimentation, investigation and data analysis (hereafter ‘practical work’).

**Figure 1.**
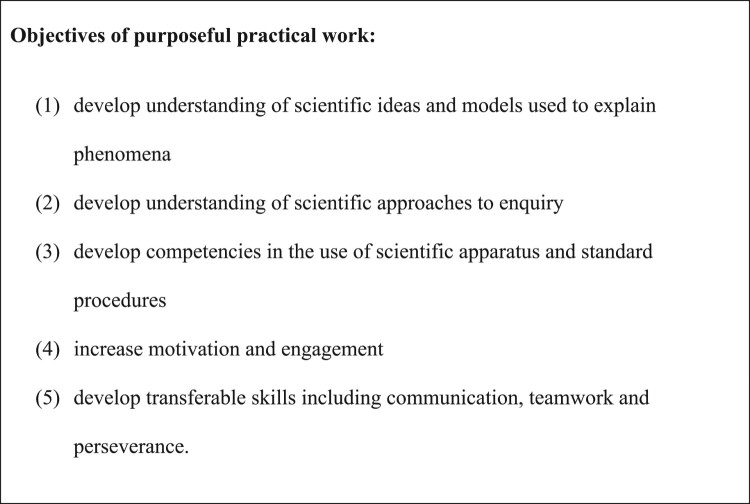
From Millar and Abrahams (2009) and Holman (2017).

The role and effectiveness of practical work in teaching and learning has been debated (e.g. Abrahams & Millar, 2008; Osborne, 2015), but various purposes of practical work in science lessons have been suggested. These include that it: makes connections between the domain of the observable or tangible and the domain of abstract ideas (Millar & Abrahams, 2009; Tiberghien, 2000); develops understanding of scientific concepts and explanations, develops scientific competencies (such as accurate observation), and develops understanding of scientific methods and epistemic insight into how scientific explanations are developed (Millar & Abrahams, 2009); fosters scientific attitudes (such as objectivity) and transferable skills (such as problem-solving), and increases motivation and engagement with science (Abrahams, 2011; Holman, 2017). There have been calls for practical work to be used purposefully in science lessons with the objectives listed in Figure 1. The first three of these objectives indicate how practical work contributes to three of the broad goals of science education (learning science, learning how science is done, and learning to do science) as defined by Hodson (2014). Pedagogical practices in practical work may be regarded as effective if they help pupils make progress towards one or more of the objectives in Figure 1.

In England, most secondary school pupils study for the General Certificate of Secondary Education (GCSE), usually awarded at age 16. Practical work has been a component of these courses since their introduction in 1986 (Childs & Baird, 2020), and is regarded as key to the development of skills that can be used in future careers (Abrahams et al., 2013). It is also seen as important for its affective aspects: a survey of over 6400 secondary school pupils in England concluded that practical work is key to motivating them in science (Hamlyn, 2020).

As discussed below, the literature reports that national assessments are a key driver of practical work pedagogy and have limiting effects on the use and effectiveness of practical work as a pedagogical tool.

### Is practical work always practical?

A study monitoring the provision of science practical work in secondary schools in England and Scotland from 2015 to 2017 defined practical work as: ‘A learning activity in which students observe, investigate and develop an understanding of the world around them, through *direct, hands-on*, experience of phenomena or manipulating real objects and materials’ (Cramman et al., 2019, p. 26, emphasis added). In practice, pupils may engage with practical work without themselves doing practical work, for example by watching a teacher demonstration or video, running a simulation, or reading a written account of the activity. Thus, pupils’ experiences of practical work are not always direct or hands-on, and pupils may develop assessable knowledge and understanding of aspects of practical work without undertaking hands-on practical work (e.g. learning by rote the sequence of steps in a practical procedure by watching a video of it).

### Practical assessment

There has been debate about what constitutes valid assessment of practical work, including the nature and range of skills and understanding that can be assessed (e.g. Black et al., 2010; Fairbrother, 1991; Gott & Duggan, 1995). Tests can assess pupils’ practical procedural knowledge and their process skills. Gott and Duggan (2002) define procedural knowledge as ‘the thinking behind the doing’, including, for example, ‘deciding how many measurements to take, over what range and with what sample, how to interpret the pattern in the resulting data and how to evaluate the whole task’ (p. 186). Process skills are the competencies needed to follow the steps in the practical procedure, which according to Hodson (1994) are ‘transferable from one context to another’ (p. 159). Abrahams et al. (2013) interpret ‘practical skills’ as a broader term that includes both procedural understanding and process skills; they differentiate between *direct* assessment of practical skills, in which a pupil’s competency is determined during practical work, and *indirect* assessment, in which a pupil’s competency is inferred from data they collected or their written account of a practical activity.

Bloom’s taxonomy of educational objectives provides hierarchies of learning objectives in three domains: cognitive, psychomotor and affective (Anderson & Krathwohl, 2001). The objectives in Figure 1 span all three of these domains, but only the first is wholly within the cognitive domain. It is difficult to assess validly outcomes in the affective domain, such as increasing pupils’ motivation and engagement with science, as these qualities can only be inferred from other behaviours (Gauld & Hukins, 1980). Measuring outcomes in the psychomotor domain (e.g. outcomes related to key process skills) requires *direct* assessment while pupils perform practical tasks. Previous analysis of assessment models has found that written practical examination questions assess only outcomes in the cognitive domain (Bennett & Kennedy, 2001). Thus, *indirect* assessment can only validly assess a subset of the objectives of practical work within the cognitive domain and does not assess objectives in the psychomotor or affective domains.

### ‘Washback’ effects of assessments on teaching

Assessments, particularly high-stakes summative assessments, have an influence on what is done in lessons (e.g. Bishop, 1995; Childs & Baird, 2020; Harlen, 2004). Assessments are considered high-stakes when the outcomes have substantial consequences for pupils (e.g. because they are the basis for certification) or for teachers and schools (e.g. because they are linked to performance measures and accountability). The merits and ethics of measurement-driven instruction have been debated (e.g. Popham, 1987; Wideen et al., 1997), but it has been found that in some cases teachers focus a significant portion of their instructional efforts on helping pupils to acquire the understanding and skills that will be tested in high-stakes assessments (e.g. Harlen, 2004; Popham, 1987).

The influence that assessments have on teaching has been described as a ‘washback’ or ‘backwash’ effect. These terms are common and well described in the literature on assessment in languages education (Cheng et al., 2015). The terms are not common in the literature on secondary school science education, but examples of washback effects of national, high-stakes summative assessments on science practical work have been described. For example, it has been found that school science teachers’ choices in their use of practical work are ‘routinely influenced’ by summative assessments (Abrahams & Saglam, 2010) and that high-stakes assessments ‘narrow the ways practical work is conducted’ in secondary schools (Childs & Baird, 2020). Abrahams et al. (2013) concluded that too great a reliance upon indirect (written) assessment ‘reduces the likelihood that practical work will be taught and learnt as well as it might be’ in schools.

Assessments clarify the specified learning outcomes of teaching by further defining and operationalising what pupils will be required to do with their understanding; this clarifies what kinds of learning experiences may be required (or sufficient) to help pupils achieve these outcomes (Millar, 2013). When an assessment has a limiting effect on what is taught, for example when a teacher does not teach a concept or competency (that they otherwise would have taught) because it will not be assessed, this an example of *negative* washback; this may be most likely to occur when there are additional pressures such as limited teaching time and budgets. When an assessment rewards, and therefore incentivises, teaching practices that would not otherwise have been adopted (including the teaching of concepts or skills that would not otherwise have been taught), this is an example of *positive* washback (Alderson & Wall, 1993). The examples of washback effects of assessments on science practical work described in the preceding paragraph are negative; examples of positive washback effects on practical work are difficult to find in the existing literature.

## Background to the study and research questions

The study was undertaken in the wake of reforms to summative assessments in science at age 16 in England. These reforms included a move from teacher assessment of pupils’ practical competencies over two years (via indirect and some direct assessment) to entirely external, indirect assessment via written questions in high-stakes, summative, terminal examination papers.

The washback effects of an assessment cannot be considered to be automatic or to manifest in the same way in every classroom because of ‘intervening factors’ including individual teachers’ levels of experience and attitudes, availability of facilities and resources, and the practices and attitudes in individual schools (Spratt, 2005). Yet washback has been said to be an inherent quality of any kind of assessment (Eckstein & Noah, 1993). Assessments are most likely to exert washback effects on the amount and types of practical work done in science lessons when they are high-stakes, summative, and when the questions assessing knowledge and understanding of practical work account for a substantial proportion^1^ of the marks used for certification (e.g. a proportion sufficient to affect the outcome by at least one grade). It is therefore important that these assessments are constructed to minimise negative washback effects on practical work in lessons (wherein it becomes limited in frequency and scope, focussed only on the assessed objectives), and maximise positive washback effects. To achieve this, the assessments must differentiate between pupils who have experienced practical work in different ways and must reward pupils who have undertaken plentiful practical work that develops a broad range of learning objectives, to incentivise such practice in classrooms.

Regarding practical work pedagogy in school science, previous studies have investigated the effectiveness of hands-on practical activities in supporting learning, with meta-analyses acknowledging heterogeneity in reported effect sizes (Caglak, 2017; Schwichow et al., 2016). It is harder to find studies that have directly compared instructional modes in school science practical work such as hands-on practical activity, teacher demonstration and video demonstration (examples include: Maričić et al., 2019; McKee et al., 2007; Moore et al., 2020); as noted by Reiss et al. (2023), ‘some, but relatively few, studies have focused on … whether practical work undertaken in particular ways associates with any educational or other outcomes’ (p3). The extent to which teachers should guide pupils or allow them to guide their own learning (such as through open inquiry) during practical work has been debated (Dobber et al., 2017; Gericke et al., 2023; Lazonder & Harmsen, 2016).

A relative dearth in empirical research on the assessment of science practical work has been reported (Abrahams et al., 2013; Childs & Baird, 2020), and a recent systematic review of research on laboratory work in secondary schools called for further research in this area (Gericke et al., 2023). This paper adds to the literature by describing findings in relation to three research questions:

1. Can written examination questions differentiate between (by differentially rewarding) pupils who have completed practical activities in different ways?
2. What are the generalizable characteristics of written questions that differentiate in this way?
3. What are the generalizable characteristics of practical work pedagogy associated with better performance on written questions that differentiate in this way?

We conclude by considering ways in which high-stakes, written assessments could be constructed to better incentivise some pedagogical practices in science practical work, and what these practices may include.

## Methods

### Overview of study design

This empirical, mixed-methods study was conducted over 3.5 years between 2018 and 2021, in accordance with ethical guidelines (BERA, 2018).

Data collection focussed on practical activities common in secondary school science courses (Table 1). Four interventions were compared for each activity:

- doing a hands-on practical version of the activity
- watching a teacher demonstration of the activity
- watching a video demonstration of the activity
- reading a description of the activity.

**Table 1.** The practical activities subject to interventions.

Practical activity	Associated practical skills
Quadrat sampling (fieldwork)	Measuring distribution and abundance of organisms
Making salt (copper sulfate)	Reacting to excess, separation, crystallisation
Reaction of sodium thiosulfate and hydrochloric acid	Measuring rate of reaction using the ‘disappearing cross’ method
Addition of masses to suspended spring	Measuring extension

For the investigation of research questions 1 and 2, quantitative data on pupil performance were collected from post-intervention tests comprising written questions. Test marks were compared to investigate the ability of the examination questions to differentiate between the pupils in the intervention groups.

For the investigation of research question 3, qualitative data on practical work pedagogy were collected from observations of intervention lessons and semi-structured interviews with the teachers.

### Sample

Participants comprised 1911 pupils aged 14–15 and their teachers in 105 science lessons. The lessons took place in 18 secondary schools in two regions of England (one in the southeast and one in the north). After participant attrition and timetabling issues in some schools, the intervention cohorts were not balanced on factors such as pupils’ pre-intervention ability, socio-economic contexts and levels of teacher experience. It was necessary to obtain balanced cohorts for comparison between the intervention groups, especially since socio-economic context and teacher experience have been shown to affect pedagogic practice in practical work (Ferreira & Morais, 2020). A ‘fair sample’ of approximately equivalent cohorts of pupils undertaking each intervention was generated for data analysis by excluding some classes on the basis of pupil characteristics (teacher-generated predicted examination grades for the pupils, indicators of socio-economic status including the percentage of pupils receiving free school meals and the percentage having English as an additional language, pupils’ ethnicities, and the percentage of pupils with special educational needs) and the length of teaching experience of the teacher. Conclusions are drawn from the resultant ‘fair sample’, comprising data from 1486 post-intervention tests completed by 1303 pupils.^2^ There were few classes with very low predicted examination grades and it was necessary to exclude these classes to generate the ‘fair sample’; thus, the analysed sample comprised pupils across a broad range of predicted grades from high to the lower end of average, but did not include pupils with the lowest predicted grades.

The ‘fair sample’ comprised classes from schools in both regions. There are differences in socio-economic factors within and between both regions, but by combining the two regions and then excluding some classes from the data set, it was possible to create a ‘fair sample’ of approximately equivalent cohorts of pupils undertaking each intervention that were balanced on socio-economic indicators and other factors. It was not possible to generate ‘fair samples’ for each region separately, as the cohorts of students in each intervention group in the resultant samples would have been too small and/or imbalanced on factors such as predicted grades (used as an indicator of pre-intervention ability).

### Interventions and post-intervention tests

Each intervention was designed to be completed within a one-hour lesson. Teachers received pupil worksheets, a presentation for use in the lesson, and instructions for conducting the intervention. The interventions were led by the teachers independently of the researchers. Teachers also received sealed packs of printed post-intervention tests to be completed by pupils in 15 min of lesson time immediately after each intervention.^3^ Teachers were not privy to the test questions prior to distributing them to pupils. The questions and mark schemes were drawn from three sets of national science examinations in England.

**Figure 2.**
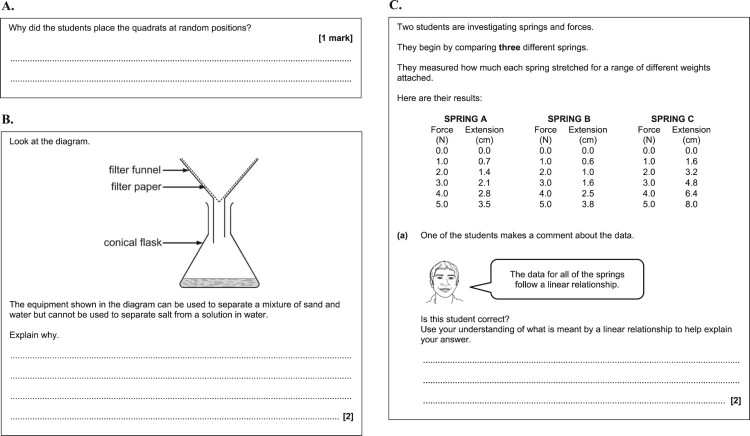
Examples of examination questions assessing different orders of thinking skills in Bloom’s cognitive domain: **A.** Recall of learned details of practical techniques and procedures. **B.** Application of practical knowledge and understanding in an unfamiliar scenario. **C.** Analysis of presented information, including interpretation, evaluation and drawing conclusions. (A: © AQA 2018. Reuse not permitted. B and C: © OCR 2018. Reuse not permitted.)

**Figure 3.**
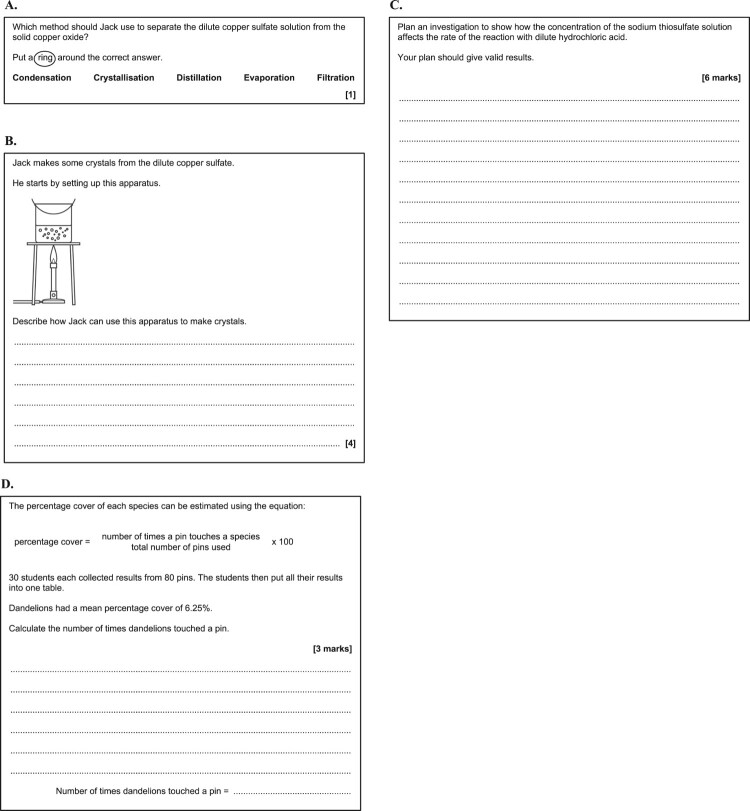
Examples of examination questions requiring different answer formats: **A.** Multiple-choice; **B.** Short answer; **C.** Extended answer; **D.** Mathematical. (A and B: © OCR 2018. Reuse not permitted. C: © AQA 2018. Reuse not permitted. D: © AQA 2019. Reuse not permitted.)

A different post-intervention test was used for each practical activity, comprising written questions pertaining to the practical activity compiled from national examination papers. For each activity, pupils in all four intervention groups sat the same test. The questions assessed knowledge and understanding of practical work across the following broad aspects: knowledge of the use of apparatus and techniques; planning of practical procedures; evaluation and improvement of practical procedures; data processing (including mathematical processing and graphical representation); and interpretation and evaluation of data. Each test comprised a mix of questions assessing different orders of thinking skills (recall, application, and analysis) within Bloom’s cognitive domain, and requiring pupils to answer in different ways (see Figures 2 and 3 for examples of these types of questions). Pupils’ responses to the tests were anonymised and blind marked by the researchers.

Pre-intervention tests were not used in this study for several reasons. The focus of the study was limited to examination questions used in high-stakes, summative science examinations at age 16 in England since 2018. When the study began in 2018 there was a limited pool of these questions from which to draw when assembling the tests, so pre-intervention tests would have had to use the same questions as the post-intervention tests. To avoid a ‘retest effect’ in which pupils performed better on the post-intervention tests as a result of having seen the questions in pre-intervention tests (irrespective of the intervention), pre-intervention tests were not used. External to the study, neither science teachers nor pupils have foresight of the practical-themed written questions in the summative examination papers while they are undertaking practical work in lessons, so replicating this arrangement improves the external applicability of our conclusions. Teacher-generated predicted examination grades for the pupils were used in lieu of pre-intervention tests to make assumptions about pupils’ pre-intervention abilities.

It was not possible to obtain statistics from the awarding organisations on how written assessment items included in the post-intervention tests performed in the national assessments from which they were derived (e.g. facility and discrimination indices), so these measures could not be considered in the analysis presented in the Results section.

### Quantitative data analysis

Quantitative analysis investigated the ability of the examination questions to differentiate between (by differentially rewarding) the pupils in the intervention groups.

#### Comparison of marks for the same practical activity

To compare the effect of intervention type on the marks achieved for a particular question (or for a group of questions) on one practical activity, a one-way ANOVA test and appropriate follow-up tests were performed. The marks for most questions, and groups of questions, were not normally distributed but the sample size was large enough for this not to invalidate the analyses. As a check, a Kruskal–Wallis test for non-parametric data was used to confirm any statistically significant differences. Homogeneous data sets were analysed using a classic ANOVA test, and any statistically significant differences found were followed up using Tukey HSE post-hoc tests to compare the mean marks in one-to-one comparisons of each possible pair of intervention groups. For non-homogeneous data a Welch’s ANOVA test was used and followed up with Games-Howell post-hoc tests. The Cohen’s d effect size of each statistically significant difference was calculated (Cohen, 1988) and reported with qualitative descriptors (Sawilowsky, 2009).

#### Comparison of marks across different practical activities

The questions in the post-intervention tests were different for each practical activity, so when grouping questions from different practical activities together an ANOVA test could not be used to compare the effect of intervention type on the mean mark achieved. The combined mean mark and 95% confidence interval were compared instead. The mean marks were deemed to be significantly different where there was no overlap in the 95% confidence intervals. Mean marks and variations were used to calculate the Cohen’s d effect sizes between pairs of intervention groups.

### Lesson observations and teacher interviews

In-person observations of 28 of the 105 intervention lessons were undertaken, and semi-structured interviews were conducted with 31 of the teachers. The interviews were audio recorded and transcribed.

### Qualitative data analysis

Qualitative analysis characterised pedagogy associated with the four interventions and identified aspects of pedagogy associated with better pupil performance on the post-intervention tests.

Field notes from lesson observations and transcripts of semi-structured interviews were scrutinised to identify common themes in the accounts of how the intervention lessons were conducted, including examples of teacher practices and pupil behaviours.

## Results and discussion

### Results pertaining to research question 1: can written examination questions differentiate between (by differentially rewarding) pupils who have completed practical activities in different ways?

**Figure 4.**
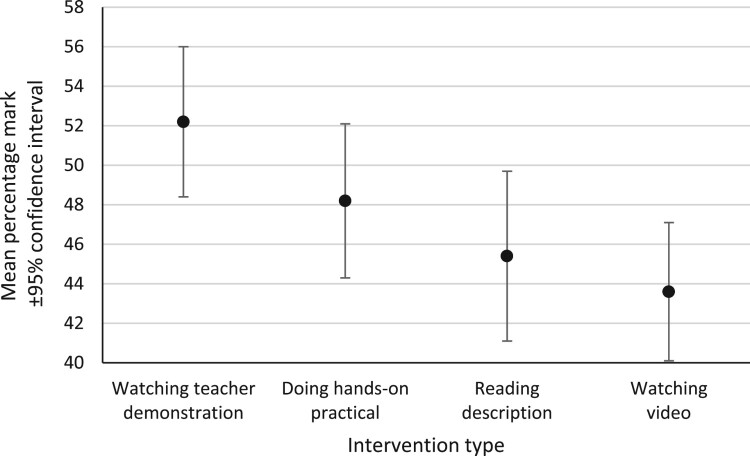
Mean whole-test marks achieved by pupils on the post-intervention tests (all practical activities combined for each intervention type).

To provide an initial, broad analysis at whole-test level, the post-intervention test scores from all the practical activities were collated to provide a combined data set for each intervention group. The mean whole-test percentage mark achieved by the pupils was calculated for each group (Figure 4) and compared. There were differences in these mean marks for all four groups, but the only statistically significant difference was between the teacher demonstration intervention (52.2% ± 3.8%; mean mark ±95% confidence interval) and the video intervention (43.6% ± 3.5%); large effect size (0.99; Cohen’s d). Mean marks for the hands-on practical intervention (48.2% ± 3.9%) and the reading intervention (45.4% ± 4.3%) were not significantly different to any other intervention group.

However, the tests comprised a mix of questions assessing different orders of thinking skills and requiring pupils to answer in different ways. Further analyses investigated the abilities of subsets of questions with different characteristics to differentiate between the intervention groups, as follows.

### Results pertaining to research question 2: what are the generalizable characteristics of written questions that differentiate in this way?

#### Difficulty and mark tariff

As a measure of question difficulty, the facility of each question was calculated by dividing the mean mark by the maximum possible mark. A higher facility indicates that a greater proportion of the cohort answered the question correctly, suggesting they did not find it difficult. The questions from all the post-intervention tests were ranked according to their facility, and the percentage of questions that differentiated between the intervention groups in each quartile of the rank order was determined (Table S1 in supplemental material). Separately, the questions were also ranked according to their mark tariff (the maximum achievable mark for each question; Table S2).

Questions with a lower facility (higher difficulty) were better at differentiating between the intervention groups. Most of the questions in the fourth facility quartile (lowest difficulty) were answered with full marks by a high proportion of pupils, so differentiation between the intervention groups was not achieved (suggesting that none of the interventions conferred a greater or lesser advantage in answering the questions). Questions with a higher mark tariff were better at differentiating between the intervention groups.

#### Questions assessing different orders of thinking skills

**Figure 5.**
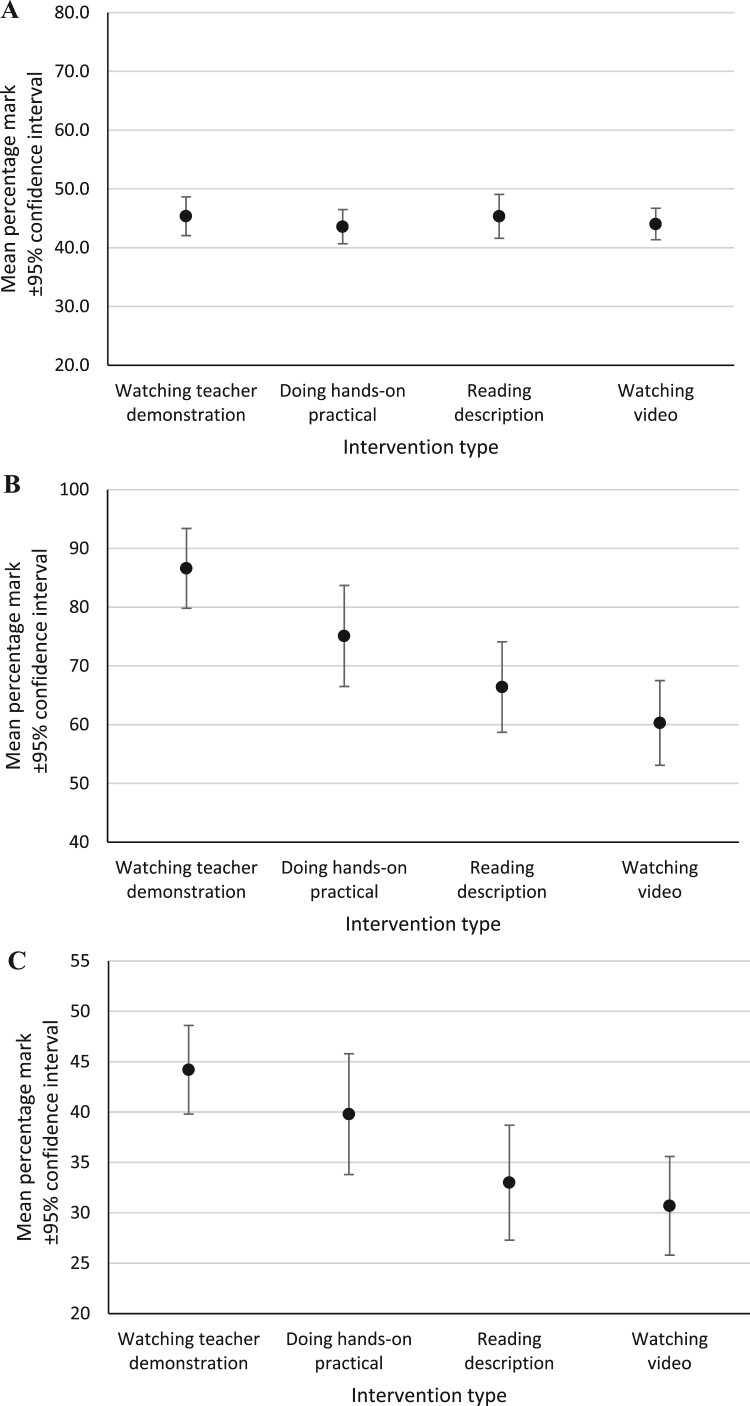
Mean percentage mark achieved by pupils on the post-intervention tests (all practical activities combined for each intervention type). **A.** Questions assessing recall. **B.** Questions assessing application. **C.** Questions assessing analysis.

*Recall:* When considering all the questions assessing pupils’ ability to recall learned details of familiar practical techniques and procedures, there were no statistically significant differences between the mean percentage marks achieved across the intervention groups (Figure 5A).

Thus, as a set, questions assessing recall did not differentiate effectively between the intervention groups. However, some individual questions assessing recall did differentiate; these are described below in the sections presenting results for multiple choice questions and questions requiring a short written answer.

*Application:* When considering all the questions assessing pupils’ ability to apply their practical knowledge and understanding in an unfamiliar scenario, there were statistically significant differences in the mean percentage marks achieved for the teacher demonstration intervention (86.6% ± 6.8%) compared with the reading intervention (66.4% ± 7.7%), large effect size (1.02); and the teacher demonstration intervention compared with the video intervention (60.3% ± 7.2%), very large effect size (1.26) (Figure 5B). The mean mark for the hands-on practical intervention (75.1% ± 8.6%) was not significantly different to any other intervention group.

Thus, as a set, questions assessing application were better at differentiating between the intervention groups than questions assessing recall. The differentiation achieved by application questions requiring pupils to answer in particular ways is discussed below.

*Analysis:* When considering all the questions assessing pupils’ ability to analyse presented information and data to interpret, evaluate and draw conclusions from it, there were statistically significant differences in the mean percentage marks for the teacher demonstration intervention (44.2% ± 4.4%) compared with the reading intervention (33.0% ± 5.7%), medium effect size (0.77); and the teacher demonstration intervention compared with the video intervention (30.7% ± 4.9%), large effect size (0.95) (Figure 5C). The mean mark for the hands-on practical intervention (39.8% ± 6.0%) was not significantly different to any other intervention group.

Thus, as a set, questions assessing analysis differentiated between the intervention groups, though not as powerfully as questions assessing application (for which the effect sizes were larger).

The finding that questions assessing application or analysis differentiated better between the intervention groups than questions assessing only recall was also generally true when subsets of questions requiring particular styles of answer were analysed, as follows.

#### Multiple-choice questions

There were four multiple-choice questions in the post-intervention tests. Two of these did not differentiate between the intervention groups, as determined by a one-way ANOVA, specifically: a question testing recall of how to read a measurement from a meniscus (F(3,477) = 1.351, *p* = 0.257); and a question testing recall of the name of the technique used to separate excess solid reactant from a solution (F(3,477) = 1.707, *p* = 0.165).

The other two multiple-choice questions differentiated between the intervention groups. One of these assessed recall of an aspect of safe working (how to move a lit Bunsen burner safely). Better differentiation, as determined by a one-way Welch’s ANOVA (F(3,448) = 10.303, *p* = <0.001), was achieved by a question with lower facility (higher difficulty) that assessed pupils’ ability to apply their understanding to identify dependent and independent variables in an experiment (Table S3).

#### Questions requiring an extended written answer

Four of the questions in the post-intervention tests required an extended answer. One of these questions differentiated between the intervention groups with statistical significance as determined by a one-way Welch’s ANOVA (F(3,233) = 6.591, *p* = <0.001). The mean mark for the teacher demonstration intervention was significantly higher than for each of the other intervention groups (Table S4A in supplemental material). This question assessed all three orders of thinking skills (recall, application, analysis). It required pupils to apply their knowledge and understanding of fieldwork techniques to describe how the population sizes of plants in a given area of grassland could be investigated, and to describe the processing of the data.

The three other questions requiring an extended answer assessed only recall (of the procedure learned for the practical activity during the intervention). There were no statistically significant differences in the mean marks across the intervention groups for these three questions (Table S4B).

#### Questions requiring a short written answer

When all the short-answer questions that assessed only recall were grouped, there were no statistically significant differences between the mean marks across the intervention groups (Table S5). Further analysis revealed that individual questions of this type did not differentiate between the intervention groups when they assessed only recall of methodological steps – i.e. recall of *what* was done. However, individual questions of this type did differentiate between the intervention groups (as determined by a one-way Welch’s ANOVA and Games-Howell post-hoc tests, with mostly medium effect sizes) when they assessed recall of not just what was done but *why* it was done, for example to increase accuracy or safety (Table 2).

**Table 2.** Examples of knowledge assessed by questions requiring a short written answer (SWA) and assessing only recall.

Knowledge assessed by SWA questions that did not differentiate between the intervention groups:	Knowledge assessed by SWA questions that differentiated between the intervention groups:
Recall steps of the procedure to make salt crystals by evaporation Recall steps of the procedure to separate excess solid reactant from solution by filtration Recall steps of the procedure to measure extension of a spring Recall that it takes more force to stretch a stiffer spring Recall steps of the procedure to correct a zero error on a newton meter	Recall which apparatus to use to measure volume *more accurately* Recall ways of working *safely* when measuring out acid Recall *why* a reactant is added to excess Recall *why* evaporating over a water bath is *safer* than over a flame

A nuanced picture also emerged from short-answer questions that assessed application or analysis. When all these questions were grouped, there were several statistically significant differences in the mean marks achieved across the intervention groups (Table S6). Thus, as a set, short-answer questions assessing application or analysis did differentiate between the intervention groups. However, further analysis revealed that individual questions of this type did *not* differentiate between the intervention groups when the answers could be deduced by interpreting a provided diagram. For example, one such question provided diagrams of a suspended spring before and after a mass was attached and asked pupils to state two ways in which the appearance of the spring had changed. Differentiation between the intervention groups was not achieved because the expected answers (e.g. that the spring had increased in length or had wider gaps between the coils after the mass was attached) could be deduced from the diagrams, and none of the interventions appeared to confer a greater or lesser advantage in answering the question.

#### Mathematical questions

Questions requiring pupils to use mathematical skills typically assess application and analysis. When all the mathematical questions were grouped, there were several statistically significant differences in the mean percentage marks achieved across the intervention groups (Table S7). Thus, as a set, mathematical questions differentiated between the intervention groups. Further analysis revealed that two of the questions did *not* differentiate:

The first was a 1-mark question with high facility (low difficulty), in which pupils had to count plants in a diagram of a quadrat and state the total number of individual plants of each species. Differentiation was not achieved because most pupils got it correct, and none of the interventions appeared to confer a greater or lesser advantage in answering the question.

The other question was a 4-mark calculation of the gradient of a tangent to a curve on a graph. The graph showed data that could be used to calculate the rate of reaction of sodium thiosulfate with hydrochloric acid. McAlinden and Noyes (2019) offer relevant insight into why this question did not differentiate between the intervention groups. In their analysis of questions assessing mathematical skills in national science examinations taken at age 18, they considered the degree to which the mathematics was ‘embedded’ within the science subject content, or – as they also put it – the degree of ‘entanglement’ between the mathematics and the science. They considered a question with low embedding/entanglement to be one that could be answered with only mathematical skills and little or no knowledge of the science; and a question with high embedding/entanglement to be one in which it would be difficult or impossible to access the mathematical work without understanding of the science. Calculating the gradient of a tangent is a mathematical skill that pupils in chemistry and biology lessons usually rehearse in the context of calculating the rate of a chemical reaction. In the question we tested, pupils were directly cued to use the rehearsed mathematical techniques – they were not asked to use the graph to calculate the rate of the reaction, but to calculate the gradient of a tangent to the curve on the graph; thus, the question had very low entanglement with the practical context. When answering this question, none of the interventions appeared to confer a greater or lesser advantage. Pupils with ample experience of calculating gradients of tangents would be able to score full marks without applying understanding gained from having done the experiment.

The mathematical questions in the post-intervention tests that did differentiate between the intervention groups generally had higher degrees of entanglement with the practical context. Why might experience of practical work confer an advantage in answering these questions? Redish and Kuo (2015) note that many pupils struggle with the use of mathematics to make meaning in science. For example: in science the numbers and symbols used in equations represent physical quantities and experimental variables; and in science the result of a calculation can be the means to developing an explanation for a real-world phenomenon. Redish and Kuo suggest that for many pupils the first step in teaching how to use calculations in science needs to be exploring the physical meanings of the numbers and symbols, which can later be tied to the mathematical procedures. Practical work can achieve this by connecting the observable/tangible and abstract domains.

### Results pertaining to research question 3: what are the generalizable characteristics of practical work pedagogy associated with better performance on written questions that differentiate in this way?

Consistently across the sets of questions analysed, the highest mean mark was achieved by pupils who experienced the teacher demonstration intervention. For most sets of questions the second-highest mean mark was for the hands-on practical intervention, and the lowest was for the video intervention. The differences between the mean marks for the teacher demonstration and hands-on practical intervention groups were not statistically significant for any of the question sets. In some sets there were significant differences between the teacher demonstration or hands-on intervention groups and the video or reading intervention groups (Table 3).

**Table 3.** Rank order of interventions.

Question set	Rank order of intervention groups by mean mark	Statistically significant differences (*p* < 0.05)
Whole tests	D > H > R > V	D > V
All recall questions	D > R > V > H	None
All application questions	D > H > R > V	D > R; D > V; H > V
All analysis questions	D > H > R > V	D > R; D > V
All extended written answer questions	D > H > R > V	None
All short written answer questions	D > H > V > R	D > R
All multiple-choice questions	D > R > V > H	None
All calculation questions	D > H > R > V	D > R; D > V; H > R; H > V

D = teacher demonstration of the practical activity; H = undertaking a hands-on version of the activity; R = reading a written description of the activity; V = video demonstration of the activity.

In qualitative data from teacher interviews and lesson observations, some aspects of pedagogy appeared to be more common in those practical experiences that were better rewarded by the test questions. An emergent theme in teacher interviews was self-reported use of teacher-led interactions such as teacher-pupil dialogue and focussed questioning during teacher demonstration interventions. An associated theme was teachers’ belief that these practices engaged pupils and encouraged them to think about what was being done and why. For example:

As I’m doing the demo, I’m explaining it to them, questioning them at the same time. (Teacher 14)

When you do the demonstration, obviously you’ve got your class and you’re getting them to think with the questioning. (Teacher 27)

[During the demonstration] we had a good discussion about what we were doing and why, and I think that allowed them to think. (Teacher 6)

If I want to really focus them and say, ‘Look, these are the key points’, I think demonstrating [is best]. I can focus their minds and get them to think about the important bits of the practical and why they’re doing it. (Teacher 18)

The final quote illustrates teachers’ use of these practices to guide pupils through key points of the practical activity, challenging them to actively think about and discuss important points of procedural understanding (‘the thinking behind the doing’) and key process skills. The use of these practices was less commonly self-reported in association with the other intervention types. This correlated with lesson observations, in which these practices were more noticeable in teacher demonstration interventions than in the other intervention types. During hands-on practical interventions more of the interactions were between pupils and the quality of pupil-pupil dialogue varied from focused and insightful to irrelevant and distracted. Some hands-on classes worked from the written practical procedure like a ‘recipe’ and this was not supplemented with sufficient elements designed to challenge pupils’ thinking; such work was busy and hands-on, but not necessarily what Abrahams (2017) and others call ‘minds-on’. The focus was more on completing procedures promptly and collecting data within the time allowed, and less on thinking about the procedural knowledge and process skills that the practical activity was intended to develop. The video interventions were more likely to be shown to the class without any teacher guidance or teacher-led interactions, rendering pupils passive observers, though this was not always the case.

Teachers with a range of teaching experience (1–39 years) were included in the study and permitted to run the interventions with relative autonomy based upon provided instructions. The instructions described the practical procedure but did not prescribe how teachers should interact with pupils. This enabled us to observe a range of pedagogical approaches and to identify aspects of pedagogy that were more common or more noticeable and appeared to be associated with higher (or lower) scores on the post-intervention tests. As these approaches were identified from a range of teacher practice, rather than being directed by the researchers, they may be recognisable to teachers outside of this study and therefore be more easily generalizable (implemented externally). Length of teacher experience was one of the variables used to assign classes to intervention groups to achieve approximately balanced cohorts, and the large sample size helped to mitigate the effects of individual teachers’ experience levels on the differences in mean test scores across the groups.

The finding that teacher guidance and teacher-led interactions were important to the effectiveness of practical work in supporting learning echoes similar reports in the literature. A systematic review of research on laboratory work in secondary schools by Gericke et al. (2023) found that teacher-guided forms of practical inquiry that include strategies such as guiding counter-questioning by the teacher in response to pupils’ ideas offer ‘better learning opportunities’ and ‘unbeatable opportunities for formative feedback’, encourage pupils to ‘regularly reflect on the relevance [of the practical work] for the subject content and learning goals’, and that teacher support of pupils ‘appears to be essential’ during practical work (p18, p31). Including organised discussions during practical work may strengthen opportunities for pupils to make meaning from the activity (Kind et al., 2011). Practical experiences that are teacher guided rather than self-directed and that have more time allocated to teacher-led discussion are associated with improved performance in written tests (e.g. Aditomo & Klieme, 2020; Eckes & Wilde, 2019; Ellwood & Abrams, 2018; Fung & Lui, 2016). Earlier work found that without careful teacher guidance during practical work pupils often became distracted by practical procedural matters at the expense of developing their conceptual understanding (e.g. Abrahams & Millar, 2008; Barker & Carr, 1989). It has been argued that much of what pupils learn from practical work may arise from discussion of what they have done rather than from the doing itself (e.g. Gunstone, 1991; Sutton, 1992). Hodson (2014) argued that learning to ‘do science’ (developing procedural knowledge and process skills) through practical work necessitates phases of teacher modelling for pupils, teacher-guided practice by pupils, and independent application by pupils; Hodson emphasised the role of the teacher in the first two phases, suggesting that teacher and pupils are ‘co-investigators’ and that pupils ‘are enabled to achieve, with judicious teacher assistance and support, a level of performance they could not achieve unaided’ (p2547).

## Conclusions

### Conclusions related to the research questions

It has been noted in the research literature that high-stakes, summative assessments influence (have ‘washback’ effects on) practical work pedagogy (Abrahams et al., 2013; Abrahams & Saglam, 2010; Childs & Baird, 2020). Hence, it is important that when such assessments are designed to test the skills and understanding developed through science practical work, they are constructed to reward – and therefore incentivise – effective pedagogical practices in practical work. To do that, the assessments must be able to differentiate between (by differentially rewarding) pupils who have experienced practical work in different ways. It is also important that we begin to define which pedagogical practices we deem to be ‘effective’ at supporting learning, and therefore worthy of being incentivised.

#### Research question 1: can written examination questions differentiate between (by differentially rewarding) pupils who have completed practical activities in different ways?

At whole-test level there were differences in the mean marks achieved by the pupils in the four intervention groups on the written examination questions in the post-intervention tests. Differentiation between the groups was weak and the only statistically significant difference was between the group who had watched a teacher demonstration (who scored highest) and the group who had watch a video demonstration. The tests comprised a mix of examination questions assessing different orders of thinking skills and requiring pupils to answer in different ways, and further analyses identified characteristics of questions that were better able to differentiate between the intervention groups, as follows.

#### Research question 2: what are the generalizable characteristics of written questions that differentiate in this way?

This study identifies generalizable characteristics of written assessment items that were more likely to differentiate between (by differentially rewarding) pupils who had experienced a practical activity in different ways (via hands-on work, watching a teacher demonstration or video demonstration, or reading a description of the activity). These characteristics include:

- assessing application (of practical knowledge and understanding in an unfamiliar scenario) and analysis (of presented information and data, including interpretation, evaluation and drawing conclusions from it), rather than just recall (of learned details of familiar practical techniques and procedures)
- requiring pupils to apply their practical experience and understanding to go beyond what is presented in a provided diagram, rather than deducing the answers entirely from the diagram
- when assessing recall, testing recall of reasons why practical procedural steps were undertaken (for example, to increase safety or accuracy of measurements), not just recall of what was done
- ‘entangling’ or embedding the assessment of mathematical skills within the practical context, requiring pupils to apply their practical experience and understanding to help solve the problem rather than relying solely upon abstract mathematical procedures.

#### Research question 3: what are the generalizable characteristics of practical work pedagogy associated with better performance on written questions that differentiate in this way?

The findings of this study indicate that written questions with the aforementioned characteristics were more likely to reward pupils who had experienced practical work with the following generalizable characteristics:

- the teacher *guided* pupils through key aspects of the practical activity (rather than leaving them to follow a ‘recipe’-style method or watch a video without teacher input), highlighting and challenging them to think about and discuss important points of procedural understanding (‘the thinking behind the doing’) and key process skills
- this prompted pupils to be *active* participants, encouraged through teacher-led interactions including teacher-pupil dialogue and focussed questioning to think and talk about what was being done and why, and relate it to their other practical experiences and existing understanding.

Practical experiences that were more guided and active in this way appeared to be better at supporting the kind of learning assessed by the questions with the aforementioned characteristics, as these experiences appeared to confer an advantage to the pupils in answering these questions.

Specific examples of teacher behaviours observed during practical work that was more guided and active, and ways in which these may support the development of assessable understanding, are given in Table 4.

**Table 4.** Theoretical mechanism for ways in which guided and active practical work could develop understanding and improve performance in written assessments.

Examples of teacher behaviour observed during guided and active practical work	Pupil understanding more likely to be developed	Level of understanding (SOLO taxonomy)	Types of written questions upon which pupil performance could be improved
Using focussed questioning and teacher-led dialogue around procedural steps.	Procedural understanding of why particular procedural steps are done (e.g. for reasons of safety or accuracy).	Unistructural and multistructural	Questions assessing recall of reasons why particular steps are done during a practical procedure.
Discussing commonalities and connections with other practical activities.	Understanding of transferable process skills (e.g. taking accurate measurements).	Relational	Questions assessing application of understanding in unfamiliar practical scenarios.
Using focussed questioning and teacher-led dialogue to relate abstract quantities and variables to observable materials and measuring instruments.	Understanding of how mathematical concepts and processes are related to and can be applied in practical contexts (transferable process skills).	Relational	Questions assessing ability to draw together their practical experiences and their mathematical skills to interpret data or perform a calculation.
Providing opportunities for pupils to identify and correct deliberate mistakes or shortcomings in a practical procedure.	Procedural understanding of how to plan and evaluate practical work.	Extended abstract	Questions assessing ability to plan or evaluate an experiment or suggest improvements.

### Implications for teaching practice and further research

It has been suggested that ‘much more must be done to assist teachers in engaging their students in school science laboratory experiences in ways that optimize the potential of laboratory activities as a unique and crucial medium that promotes the learning of science concepts and procedures, the nature of science, and other important goals in science education’ (Lunetta et al., 2007, p. 433). Although there are dangers in generalising, teachers need clear messages from research if they are to implement research-informed changes in practice. Previous studies have recognised the challenges and importance of developing science teachers’ awareness of emerging recommendations for the planning and teaching of practical work (e.g. Abrahams & Reiss, 2012; Akuma & Callaghan, 2019), and of providing training and support for their subsequent decision-making processes (de Winter & Millar, 2023; Dillon, 2010). There have been calls for science practical work to be purposeful (Hart et al., 2000; Holman, 2017; Holman & Yeomans, 2018; Millar & Abrahams, 2009), with the learning objective(s) of each activity (such as those in Figure 1) clear to both teacher and pupils. In light of our findings, we expand upon these calls by recommending that practical work be guided, active *and* purposeful (‘GAP’).

Although pupils who had watched a teacher demonstration scored highest, on average, on the written questions in this study, there were no statistically significant differences between the mean marks of the teacher demonstration and hands-on practical groups for any of the sets of questions analysed. Significant differences were observed when these marks were compared with those of pupils who had only watched a video or read a description of the activity, which were lower on average; we ascribe these differences to the increased likelihood of teacher demonstrations and hands-on practical activities being done in guided and active ways. We previously reported key points of good practice observed during teacher demonstration intervention lessons (Moore et al., 2020).

Although each practical intervention was completed within a one-hour lesson, in general the hands-on practical and teacher demonstration interventions took up more of the lesson time than the reading and video interventions. In general, pupils’ post-intervention test scores were higher after the hands-on practical and teacher demonstration interventions than after the reading and video interventions. This suggests that both the ‘format’ (i.e. hands-on practical work, teacher demonstration, video demonstration, or reading) and duration of the intervention could have affected pupils’ performance on the post-intervention tests. Evidence from lesson observations and teacher interviews suggested that both these factors were less important than the ways in which the teacher and pupils behaved during the intervention activity. Hands-on practical work was more likely to contain periods of activity (such as organising pupils, arranging or tidying up apparatus, or unguided pupil-pupil talk) that extended the duration but did not necessarily support learning.

Any practical work, regardless of the ‘format’ or duration, may be facilitated by a teacher in ways that are more or less effective in helping pupils make sense of what they are doing and make meaning by relating the practical activity to other experiences and concepts. Picking a particular ‘format’ or duration does not guarantee effective learning, as this is strongly contingent upon factors including the teacher’s and pupils’ behaviours and levels of engagement during the practical work. Variation and nuance in pedagogy were observed within all four intervention groups across the practical activities in this study. The pedagogical approaches identified through research question 3 were more often observed or reported by teachers in those practical experiences that were better rewarded by the post-intervention tests; we suggest that it was these approaches that gave the practical work the generalizable characteristics of being teacher-guided and pupil-active, and supported more effective learning (i.e. better supported pupils to achieve the learning outcomes assessed by the written questions in the post-intervention tests). Steps could be taken to imbue any type of practical work with more of these characteristics; for example, watching a video could be punctuated with teacher-led interactions that encourage pupils to be focussed, actively-thinking participants rather than passive observers. Incorporating approaches to make practical work more teacher-guided and pupil-active could increase its duration, and contextual limitations on teachers’ decision making (such as scheduling and time constraints) have been acknowledged (Puttick et al., 2015); further research could investigate whether there are measurable learning benefits associated with intentionally implementing these approaches, and explore teachers’ support needs and their perceptions of the relative benefits and demands of doing so.

In some cases a teacher demonstration is the most appropriate format for a practical activity (e.g. for reasons of safety, or due to the costs or availability of materials and apparatus), but this and the results of this study do not justify replacing all hands-on practical work with teacher demonstrations; it has been acknowledged that lack of hands-on practical experience could disadvantage pupils who need direct, first-hand experience of practical activities to better understand what is happening, including but not limited to those with visual impairment and other special educational needs (Ofqual, 2020). Some of the objectives of practical work in Figure 1, such as developing competencies in the use of scientific apparatus, can only be fully met through hands-on work. In interviews, teachers reported using in their general practice (outside of this study) combinations of teacher demonstrations, hands-on practical activities and video demonstrations to develop and consolidate procedural knowledge and process skills, and their belief that this better supported learning. Learning benefits of combining video demonstrations with hands-on practical work have been reported in the research literature (e.g. Solé-Llussà et al., 2022). For all these reasons, we recommend that teachers provide pupils with a mix of hands-on and other practical experiences across a course of study, and that each experience is planned such that it is purposeful and includes ample teacher-led interactions to ensure that it is guided and that pupils are actively-thinking participants.

The lesson observations and teacher interviews conducted in this study provided insights into teachers’ and pupils’ behaviours associated with the interventions, and teachers’ perceptions of their experiences. However, it was beyond the scope of this study to investigate pupils’ perceptions of the different interventions, which could be the basis of further investigation.

### Implications for assessment practice and further research

According to the SOLO taxonomy model (Biggs & Collis, 1982), learners’ understanding develops through various levels: unistructural, multistructural, relational, and extended abstract. This model could help to explain why the pedagogical approaches identified in this study supported pupils to better answer particular types of questions in the written assessments. Questions assessing recall are ‘declarative knowledge tests’ of unistructural and multistructural understanding, while questions assessing application and analysis are ‘functioning knowledge tests’ of relational and extended abstract understanding.

We regard the pedagogical practices characteristic of the guided and active practical work observed in this study as ‘effective’ practices for several reasons. We suggest that they help to guide pupils through the key transferable process skills in a practical activity and to actively explore key points of procedural understanding. These are points pupils may otherwise miss in a busy practical lesson in which pupils’ focus – without teacher guidance – may more naturally be on completing the prescribed procedure within the allotted time or obtaining a ‘desired’ experimental result. Practices associated with guided and active practical work may help pupils to make meaning from each practical experience, rather than seeing it simply as an exercise in working through or observing the practical procedure. This may help pupils to progress to the relational and extended abstract levels of understanding described by the SOLO model, for example by helping them to explore reasons for and commonalities between steps of practical procedures they have experienced, thus better preparing them to answer questions assessing application and analysis (Table 4).

This study was limited to investigating types of written examination questions used in high-stakes, summative science assessments in England. There are likely to be other types of written questions with generalizable characteristics that have the potential to differentiate between pupils with different experiences of practical work, which could be elucidated by further research. For now, we recommend that high-stakes, written assessments of practical knowledge and understanding are constructed to maximise the presence of questions with the characteristics identified by this study, with the intention of having positive washback effects on pedagogy by better rewarding – and therefore incentivising – practices that make practical work more guided and active, as described above.

Incentivising these practices could have benefits for pupils that include improving performance in the assessments but also supporting learning more broadly. In guided and active practical work, the teacher guides pupils’ focus towards, and encourages them to actively think about and discuss, key process skills and key pieces of procedural understanding. Some of these skills and pieces of understanding are transferable (e.g. the use of measuring instruments, and understanding issues affecting the precision and accuracy of measurements), so will be useful in further study and careers. Previous studies (e.g. Bennett & Kennedy, 2001) have found that written (indirect) assessment limits the range of practical objectives that can be assessed to those in Bloom’s cognitive domain. The results of the present study suggest that written assessments could nevertheless be constructed to reward and incentivise pedagogical practices that could make practical work more guided and active, and thus a better setting in which to develop a broader range of objectives than the written assessments can assess (e.g. key process skills).

## Supplementary Material

Supplemental Material

## Notes

1 15% in GCSE science examinations in England.

2 Some pupils completed more than one post-intervention test, and in these cases each test was completed after a different intervention type for a different practical activity.

3 In a small number of hands-on practical intervention lessons there was insufficient time to complete the post-intervention test in the same lesson, and it was completed at the beginning of the next lesson up to 3 days later. Given the short timescale and large sample size any effect on the results is likely to have been minimal.
