# Supplementary material for: Assessment and practical science: identifying generalizable characteristics of written assessments that reward and incentivise effective practices in practical science lessons
Source: Int J Sci Educ. 2023 Nov 19;46(7):643–69. doi: 10.1080/09500693.2023.2253366 (PMC11075375; doi:10.1080/09500693.2023.2253366)
Supplement: Supplemental Material [file TSED_A_2253366_SM4510.pdf]

Moore, A.M., Fairhurst, P., Bennett, J.M., Harrison, C., Correia, C.F. and Durk, J. (2022). Assessment and practical science: identifying generalizable characteristics of written assessments that reward and incentivise effective practices in practical science lessons.

Supplemental material

Table S1. Ability of questions of different facility (difficulty) to differentiate with statistical significance between the intervention groups.

Facility quartile	Percentage of questions that differentiated
1st (most difficult)	83%
2nd	60%
3rd	62%
4th (least difficult)	16%

Table S2. Ability of questions of different mark tariff to differentiate with statistical significance between the intervention groups.

Mark tariff quartile	Percentage of questions that differentiated
1st (shortest questions)	40%
2nd	60%
3rd	67%
4th (longest questions)	75%

Table S3. Post-intervention test results for a multiple-choice question assessing students' ability to apply their understanding to identify dependent and independent variables.

	One-to-one comparisons of intervention groups<br>* significant difference in mean mark ($p < 0.05$)					
	**Demo (D) vs. reading (R)**	**Demo vs. hands-on (H)**	**Demo vs. video (V) ***	**Reading vs. hands-on**	**Reading vs. video ***	**Hands-on vs. video**
**Mean mark** (Max. mark = 2)	D: 1.32<br>R: 1.10	D: 1.32<br>H: 1.00	D: 1.32<br>V: 0.73	R: 1.10<br>H: 1.00	R: 1.10<br>V: 0.73	H: 1.00<br>V: 0.73
**p value** (Games-Howell post-hoc test)	0.210	0.079	<0.001	0.899	0.008	0.178
**Effect size** (Cohen's d)	0.25<br>small	0.35<br>small	0.69<br>medium	0.11<br>very small	0.43<br>medium	0.29<br>small

Table S4. Post-intervention test results for: **A.** one extended writing question assessing recall, application and analysis; **B.** a group of three extended writing questions assessing only recall.

**A.**	**One-to-one comparisons of intervention groups<br>* significant difference in mean mark (p < 0.05)**					
	**Demo (D) vs. reading (R) ***	**Demo vs. hands-on (H) ***	**Demo vs. video (V) ***	**Reading vs. hands-on**	**Reading vs. video**	**Hands-on vs. video**
**Mean mark**<br>**(Max. mark = 6)**	D: 3.35<br>R: 2.43	D: 3.35<br>H: 2.48	D: 3.35<br>V: 2.31	H: 2.48<br>R: 2.43	R: 2.43<br>V: 2.31	H: 2.48<br>V: 2.31
**p value**<br>**(Games-Howell post-hoc test)**	0.003	0.005	<0.001	0.999	0.980	0.947
**Effect size**<br>**(Cohen's d)**	0.66<br>medium	0.72<br>medium	0.71<br>medium	0.03<br>very small	0.07<br>very small	0.10<br>very small

**B.**	**One-to-one comparisons of intervention groups<br>* significant difference in mean mark (p < 0.05)**					
	**Demo (D) vs. reading (R)**	**Demo vs. hands-on (H)**	**Demo vs. video (V)**	**Reading vs. hands-on**	**Reading vs. video**	**Hands-on vs. video**
**Mean mark**<br>**with 95% confidence interval**<br>**(Max. mark = 18)**	D: 9.37 ± 0.97<br>R: 9.24 ± 0.95	H: 9.38 ± 0.81<br>D: 9.37 ± 0.97	D: 9.37 ± 0.97<br>V: 8.89 ± 0.78	H: 9.38 ± 0.81<br>R: 9.24 ± 0.95	R: 9.24 ± 0.95<br>V: 8.89 ± 0.78	H: 9.38 ± 0.81<br>V: 8.89 ± 0.78
**Effect size**<br>**(Cohen's d)**	0.05<br>very small	0.00<br>negligible	0.19<br>very small	0.06<br>very small	0.14<br>very small	0.19<br>very small

Table S5. Combined post-intervention test results for all questions requiring a short written answer and assessing only recall.

	**One-to-one comparisons of intervention groups<br>* significant difference in mean mark (p < 0.05)**					
	**Demo (D) vs. reading (R)**	**Demo vs. hands-on (H)**	**Demo vs. video (V)**	**Reading vs. hands-on**	**Reading vs. video**	**Hands-on vs. video**
**Mean mark**<br>**with 95% confidence interval**<br>**(Max. mark = 11)**	D: 4.89 ± 0.57<br>R: 4.84 ± 0.59	D: 4.89 ± 0.57<br>H: 4.20 ± 0.48	V: 4.97 ± 0.41<br>D: 4.89 ± 0.57	R: 4.84 ± 0.59<br>H: 4.20 ± 0.48	V: 4.97 ± 0.41<br>R: 4.84 ± 0.59	V: 4.97 ± 0.41<br>H: 4.20 ± 0.48
**Effect size**<br>**(Cohen's d)**	0.03<br>very small	0.37<br>small	0.05<br>very small	0.34<br>small	0.08<br>very small	0.41<br>medium

Table S6. Combined post-intervention test results for all questions requiring a short written answer and assessing application or analysis.

	**One-to-one comparisons of intervention groups<br>* significant difference in mean mark ($p < 0.05$)**					
	**Demo (D) vs. reading (R) ***	**Demo vs. hands-on**	**Demo vs. video ***	**Reading vs. hands-on ***	**Reading vs. video**	**Hands-on vs. video**
**Mean mark** with 95% confidence interval (Max. mark = 12)	D: 5.41 ± 0.65<br>R: 3.98 ± 0.54	D: 5.41 ± 0.65<br>H: 5.29 ± 0.59	D: 5.41 ± 0.65<br>V: 4.28 ± 0.43	H: 5.29 ± 0.59<br>R: 3.98 ± 0.54	V: 4.28 ± 0.43<br>R: 3.98 ± 0.54	H: 5.29 ± 0.59<br>V: 4.28 ± 0.43
**Effect size** (Cohen's d)	0.88<br>large	0.07<br>very small	0.68<br>medium	0.84<br>large	0.19<br>very small	0.63<br>medium

Table S7. Combined post-intervention test results for all questions requiring a calculation.

	**One-to-one comparisons of intervention groups<br>* significant difference in mean mark ($p < 0.05$)**					
	**Demo (D) vs. reading (R) ***	**Demo vs. hands-on (H)**	**Demo vs. video (V) ***	**Reading vs. hands-on ***	**Reading vs. video**	**Hands-on vs. video ***
**Mean mark** with 95% confidence interval (Max. mark = 13)	D: 6.98 ± 0.74<br>R: 4.53 ± 0.81	D: 6.98 ± 0.74<br>H: 6.43 ± 1.04	D: 6.98 ± 0.74<br>V: 4.27 ± 0.72	H: 6.43 ± 1.04<br>R: 4.53 ± 0.81	R: 4.53 ± 0.81<br>V: 4.27 ± 0.72	H: 6.43 ± 1.04<br>V: 4.27 ± 0.72
**Effect size** (Cohen's d)	0.96<br>large	0.21<br>small	1.07<br>large	0.73<br>medium	0.11<br>very small	0.84<br>large
